# *FGFR1* variation in the divergent settings of congenital hypopituitarism and pituitary tumours

**DOI:** 10.1007/s11102-025-01498-0

**Published:** 2025-03-10

**Authors:** Andreas Orsmond, Gayathri Krishnan, Lyle J. Palmer, Sunita M. C. De Sousa, Ann McCormack

**Affiliations:** 1 Garvan Institute of Medical Research, 384 Victoria St, Darlinghurst, NSW 2010 Australia; 2 Hospital Kuala Lumpur, Kuala Lumpur, Malaysia; 3 School of Public Health, University of Adelaide, Adelaide, Australia; 4 Adelaide Medical School, University of Adelaide, Adelaide, Australia; 5 Endocrine & Metabolic Unit, Royal Adelaide Hospital, Adelaide, Australia; 6 South Australian Adult Genetics Unit, Royal Adelaide Hospital, Adelaide, Australia; 7 Department of Endocrinology, St Vincent’s Hospital, Sydney, NSW Australia; 8 St Vincent’s Clinical School, University of New South Wales, Sydney, NSW Australia

**Keywords:** Pituitary tumour, Pituitary adenoma, Hypopituitarism, *FGFR1*, Next generation sequencing

## Abstract

**Purpose:**

Pituitary tumours are relatively common, and familial in approximately 5% of cases. However, germline genetic contributions to pituitary tumour development are incompletely characterised. Preliminary evidence suggests pituitary tumours may be promoted by variants in pituitary organogenesis genes. Our study aimed to identify rare germline variants in pituitary organogenesis genes that may contribute to pituitary tumour development.

**Methods:**

A familial case of pituitary disease was investigated. We also examined 36 pituitary organogenesis genes in 134 individuals with pituitary tumours using a targeted next-generation sequencing panel, identifying and characterising variants with a population allele frequency < 0.05%.

**Results:**

One patient with a prolactin-secreting pituitary tumour and his daughter with combined pituitary hormone deficiency shared a rare germline variant in *FGFR1*, c.386 A > C, p.(D129A). In our broader study, we identified an additional individual with the *FGFR1* D129A variant and demonstrated enrichment compared to a control population derived from the Genome Aggregation Database (gnomAD). We also observed 66 rare germline variants in pituitary organogenesis genes amongst 54/134 individuals (40%). However, compared to control data, the study cohort exhibited no enrichment for other rare variants in *FGFR1*, FGF-related genes, or other pituitary embryogenesis genes.

**Conclusion:**

Our results suggest that the *FGFR1* D129A variant may be associated with pituitary tumorigenesis but the role of other pituitary embryogenesis genes remains unclear. Additional independent cohorts and functional studies are required.

**Supplementary Information:**

The online version contains supplementary material available at 10.1007/s11102-025-01498-0.

## Introduction

Up to 90% of individuals with familial isolated pituitary tumours lack identifiable germline pathogenic variants in canonical susceptibility genes [1, 2]. Case reports, mouse models and cell-culture models propose that genes involved in pituitary organogenesis may promote pituitary tumorigenesis [3–6], so are biologically plausible candidates to investigate for tumour-promoting germline variants.

We aimed to characterise rare germline variants in pituitary organogenesis genes that may promote tumorigenesis in individuals with familial and sporadic pituitary tumours. We first describe our study’s index kindred, in which a proband and his daughter with the same rare variant in the Fibroblast Growth Factor Receptor 1 (*FGFR1*) gene developed an early-onset pituitary tumour and combined pituitary hormone deficiency (CPHD), respectively. Subsequently, we report on the use of a next-generation sequencing (NGS) panel of 36 pituitary organogenesis genes among 134 individuals with pituitary tumours.

## Materials and methods

This study amalgamated two different study cohorts of unrelated individuals with pituitary tumours. A first cohort (*n* = 82, including the index case), the subject of an earlier publication [7], recruited patients with pituitary tumour onset before age 40 or a personal or familial history of pituitary tumour or MEN-1 related endocrine neoplasia. A second cohort (*n* = 52) was selected from individuals participating in our pituitary tumour biobank who had undergone pituitary surgery.

Germline DNA was extracted and sequenced with a custom NGS panel as previously described [8]. Sequences were aligned and variants annotated as detailed by De Sousa et al. [7]. The NGS panel included 36 genes putatively involved in pituitary organogenesis (listed in Table 1), which formed the basis of this study [9].

**Table 1:** Rare variant counts in individuals with pituitary tumours compared to controls. The number of rare variants in individuals in the pituitary tumour cohort and control cohort (from GnomAD v4.0) are recorded. Rare variant counts were compared across control and patient cohorts using the ProxECAT method, which corrects for differences in coverage using synonymous variant counts. P values before and after Benjamani-Hochberg corrections are listed. Relative risks are reported before correction for differences in coverage

Gene	Control RV count (*n* = 730947)	Patient cohort RV count (*n* = 134)	Control SV count (*n* = 730947)	Patient SV count (*n* = 134)	RR of RV exposure in patient cohort	Proxecat *p* value	Corrected *p* value
*ATR*	20,515	7	3,511,072	588	1.86	0.094	1.000
*ATRX*	10,966	3	24,741	7	1.49	0.961	1.000
*AXL*	12,169	4	15,935	3	1.79	0.463	0.979
*BCOR*	10,386	1	248,147	47	0.53	0.454	1.000
*BMP4*	5858	3	6969	1	2.79	0.233	1.000
*CHD7*	26,579	6	271,122	54	1.23	0.775	1.000
*CREBBP*	22,333	4	275,062	51	0.98	0.947	1.000
*EP300*	21,839	3	1,001,973	202	0.75	0.482	0.964
*FGF10*	1698	0	4450	2	0.00	0.256	1.000
*FGF8*	1932	2	4110	0	5.65	0.033	1.000
*FGFR1*	9767	3	49,511	11	1.68	0.630	0.945
*FGFR2*	5759	1	1,940,569	340	0.95	0.992	1.000
*FLNA*	18,148	2	224,213	18	0.60	0.684	0.984
*GATA2*	3215	2	1,027,184	155	3.39	0.105	0.945
*GLI2*	24,050	1	2,550,358	471	0.23	0.049	0.882
*GNRHR*	3676	0	127,736	10	0.00	0.451	1.000
*IGSF1*	10,239	1	1,156,890	123	0.53	0.932	1.000
*KAL1*	4583	1	4230	1	1.19	0.955	1.000
*LHX2*	2263	0	667,350	119	0.00	0.369	1.000
*LHX3*	6057	0	456,336	73	0.00	0.165	1.000
*LRP2*	49,241	11	8,614,785	1602	1.22	0.556	0.910
*MED12*	5577	1	331,860	73	0.98	0.834	1.000
*NR0B1*	3620	0	474,076	50	0.00	0.383	1.000
*OTX2*	2948	1	2941	1	1.85	0.998	0.998
*PAX5*	3703	1	2059	0	1.47	0.347	1.000
*PAX6*	2831	1	7773	3	1.93	0.938	1.000
*PAX7*	6004	2	627,759	109	1.82	0.409	1.000
*PROP1*	5342	1	672,140	118	1.02	0.950	1.000
*PTCH1*	21,608	3	611,883	118	0.76	0.553	0.949
*SHH*	3197	1	13,792	1	1.71	0.321	1.000
*SIX6*	2609	0	45,312	4	0.00	0.503	0.954
*SOX10*	3308	0	1,021,377	185	0.00	0.274	1.000
*SOX3*	1786	0	57,014	4	0.00	0.619	0.969
*SOX9*	4692	0	342,540	62	0.00	0.194	1.000
*SUFU*	5131	1	4865	2	1.06	0.530	0.953
*TCF7L1*	6191	1	41,850	8	0.88	0.871	1.000
Total	349,820	68	26,439,984	4616	1.06	0.387	
*FGFR1*,*FGF8*,*FGF10*	13,397	5	58,071	13	2.04	0.350	

Abbreviations—RV: Rare Variant, SV: Synonymous Variant, RR: Relative Risk

An in-house clinical genomics platform, *Seave*, and Integrated Genomics Viewer were used to filter variants [10, 11]. Short insertion/deletion or single-nucleotide variants without artefact and with a population allele frequency < 0.05% in gnomAD (v2.1.1) were reported [12].

Pituitary organogenesis gene variants in the gnomAD (v4.0) exome database were filtered with the same specifications as the patient cohort and used as controls. The rare variant burden in patient and control populations were statistically compared for each gene using the ProxECAT method [13] in RStudio (v4.3.2).

The number of individuals with the index kindred’s rare variant in the patient and control populations were compared using Fisher’s Exact testing.

## Results

### Index kindred

A South-Asian man was diagnosed with a giant prolactinoma at age 17 (prolactin level 88,000 mIU/L). He was initially managed with bromocriptine, with minimal tumour shrinkage. At age 19, he underwent transsphenoidal resection, fractionated radiotherapy to residual tumour, and maintenance cabergoline treatment. He subsequently emigrated to Australia, and, at last review aged 48, he is clinically well with stable residual cystic tumour and normal prolactin levels on continuing cabergoline treatment.

At age 33, he fathered a child with a consanguineous partner. His daughter developed perinatal jaundice and hypoglycaemia, with congenital strabismus, growth failure, isolated growth hormone deficiency and a hypoplastic anterior pituitary. She was later diagnosed with central hypothyroidism and hypogonadism. There is no other family history of pituitary disease, endocrine neoplasia or congenital malformation.

The proband and daughter were found to have a heterozygous variant in *FGFR1*, c.386 A > C, p.(D129A). This variant is rare, with a population allele frequency (gnomAD v4.0.0) of 0.005%. The genomics platform Varsome [14] classifies it as being of uncertain significance, as it satisfies three American College of Medical Genetics criteria for pathogenicity—PM2 (extremely rare in controls), PP2 (missense variant in a gene with low benign missense variation) and PP5 (a reputable source reports the variant as pathogenic).

No rare variants were identified in the proband on germline genetic testing of established pituitary tumour predisposition genes or general tumorigenesis genes. The daughter has not had genetic testing beyond for the familial variant. The proband’s spouse declined genetic testing.

### Pituitary tumour cohort study

Cohort demographics are available in Supplementary Table 1.

The index kindred’s *FGFR1* D129A variant was additionally found in a female South-Asian patient in the pituitary tumour cohort, also in the setting of a prolactinoma. The proportional count of *FGFR1* D129A variants was significantly higher in the patient cohort compared to gnomAD controls (*p* < 0.01). A further rare missense *FGFR1* variant (c.1447 C > T, p.(P483S), allele frequency 0.0007%), was also identified in a patient in the pituitary tumour cohort.

Sixty-six rare variants in other pituitary organogenesis genes in 54/134 individuals (40%) were identified across the patient cohort and are characterised in Supplementary Table 2. No variants were homozygous, with three hemizygous variants noted. The burden of rare variants in each pituitary organogenesis gene were not significantly enriched in individuals with pituitary tumours over controls (as detailed in Table 1), including for *FGFR1* and among FGF-related genes (*p* = 0.63 and 0.35). The same was true across the 36 pituitary embryogenesis genes collectively (RR 1.06, *p* = 0.387).

## Discussion

FGFR1 is a growth factor receptor with established roles in cell proliferation and pituitary organogenesis [15]. The index kindred’s *FGFR1* D129A variant was enriched in the pituitary tumour cohort, highlighting a potential association with pituitary tumorigenesis. The D129A variant lies within the FGFR1 acid box domain, which constitutively blocks canonical ligand binding and promotes non-canonical ligand binding [16, 17]. *FGFR1* D129A has been reported by others in an individual with Kallmann syndrome [18] which, in addition to its presence in our proband’s daughter, suggests that it may also contribute to congenital hypopituitarism. However, the proband’s daughter’s parents were consanguineous, increasing the chance that unidentified variants in alternative genes may contribute to her congenital hypopituitarism. Ultimately, functional assays are required to confirm the variant’s biological consequences.

The second *FGFR1* variant identified in a patient with pituitary tumour (*FGFR1*, c.1447 C > T, p.(P483S)) has also been found in individuals with a hypoproliferative pituitary phenotype. This variant was described in an individual with deficiency in multiple pituitary hormones in the context of septo-optic dysplasia and reduces FGFR1 signalling in vitro [19].

Taken together, our findings support the possibility that certain *FGFR1* variants may both disrupt pituitary organogenesis and promote pituitary tumorigenesis, depending on cellular context. Phenomena whereby similar germline variants produce different phenotypes have also been identified in other pituitary tumorigenesis genes, notably in *PRKAR1A* variants promoting Carney complex and acrodysosotosis [20].

However, our results suggest rare *FGFR1* variants may not all associate with pituitary tumorigenesis, as their burden was not significantly enriched in the study cohort compared to control data. This was also true of the other analysed pituitary embryogenesis genes. Several explanations are possible. Some rare variants are likely not functionally deleterious, ‘diluting’ a potential effect of true deleterious variants. It is also possible that only variants in specific regions of pituitary organogenesis genes potentiate tumorigenesis.

Study limitations include: firstly, our patient cohort sample size is small in the context of analysing rare gene variants; and, secondly, our control data were generated using different genetic platforms, reference genomes and ancestry patterns to the study cohort.

## Conclusion

We identify divergent phenotypes of childhood-onset macroprolactinoma and CPHD in a kindred with a germline *FGFR1* variant and demonstrate enrichment of this variant in individuals with pituitary tumours compared to controls. Our findings support future work analysing specific variants in *FGFR1* that may paradoxically promote pituitary developmental abnormalities and tumorigenesis, potentially providing insight into pituitary tumour pathogenesis. With further validation, this may highlight new variants/genes for inclusion in relevant gene panel testing.

## Electronic supplementary material

Below is the link to the electronic supplementary material.

Supplementary Material 1

## Data Availability

The majority of original data generated and analyzed during this study are included in this published article. Some datasets generated in this study are not publicly available but are available from the corresponding author on reasonable request.
