# Supplementary material for: FGFR1 variation in the divergent settings of congenital hypopituitarism and pituitary tumours
Source: Pituitary. 2025 Mar 10;28(2):39. doi: 10.1007/s11102-025-01498-0 (PMC11893631; doi:10.1007/s11102-025-01498-0)
Supplement: Supplementary file 1 — Supplementary Material 1 [file 11102_2025_1498_MOESM1_ESM.docx]

**Article Title***: FGFR1* variation in the divergent settings of congenital hypopituitarism and pituitary tumours

**Journal name:** *Pituitary*

**Author names and affiliations:** Andreas Orsmond^1^, Gayathri Krishnan^2^, Lyle Palmer^3^, Sunita M. C. De Sousa^4-6^, Ann McCormack^1,7,8^

^1^ Garvan Institute of Medical Research, Sydney, NSW, Australia

^2^ Hospital Kuala Lumpur, Kuala Lumpur, Malaysia

^3^ School of Public Health, University of Adelaide, Adelaide, Australia

^4^ Adelaide Medical School, University of Adelaide, Adelaide, Australia

^5^ Endocrine & Metabolic Unit, Royal Adelaide Hospital, Adelaide, Australia

^6^ South Australian Adult Genetics Unit, Royal Adelaide Hospital, Adelaide, Australia

^7^ Department of Endocrinology, St Vincent’s Hospital, Sydney, NSW, Australia

^8^ St Vincent’s Clinical School, University of New South Wales, Sydney, NSW, Australia

**Corresponding author:** Prof Ann McCormack, Garvan Institute of Medical Research, 384 Victoria St, Darlinghurst NSW Australia 2010, E: [a.mccormack@garvan.org.au](mailto:a.mccormack@garvan.org.au), ORCID 0000-0002-2859-5566

**Supplementary Table 1:** *Demographics of pituitary tumour cohorts.*

| Patient number | Female | Age at diagnosis (mean) | GH | PRL | NF (%) | ACTH (%) | TSH (%) | Gn (%) | PH (%) |
| --- | --- | --- | --- | --- | --- | --- | --- | --- | --- |
| 134 | 74 (56%) | 39 | 44 (33%) | 38 (28%) | 23 (17%) | 19 (14%) | 3 (2%) | 2 (1%) | 5 (4%) |

*Abbreviations—*GH: growth hormone secreting tumour, PRL: prolactin-secreting tumour, NF: non-functioning tumour, ACTH: Adrenocorticotropic hormone-secreting tumour, TSH: thyroid stimulating hormone-secreting tumour, Gn: gonadotropin-secreting tumour, PH; plurihormonal tumour (expressing two or more pituitary hormones)

**Supplementary Table 2:** *Rare variants identified in individuals with pituitary tumours within pituitary organogenesis genes.* Variants were classified as rare if they have a population allele frequency less than 0.05 in gnomAD v2.1.1. Published associations were derived from OMIM, HGMD and ClinVar databases [1-3]. Individuals are given an arbitrary number to show cases where they had more than one variant.

| **Gene** | **Location** | **Allele frequency (%)** | **Published Associations** | **Patient** | **Tumour Type** |
| --- | --- | --- | --- | --- | --- |
| *ATR* | c.2638G>A, p.(A880T) | 0.0060 | - | 1 | NFA |
| *ATR* | c.3497A>G, p.(H1166R) | 0.01 | - | 2 | PRL |
| *ATR* | c.4405A>G, p.(T1469A) | 0.0414 | - | 3 | PRL |
| *ATR* | c.523A>G, p.(M175V) | 0.0008 | - | 4 | ACTH |
| *ATR* | c.5522G>A, p.(R1841K) | 0 | - | 5 | NFA |
| *ATR* | c.6023G>T, p.(R2008L) | 0.0287 | - | 6 | PRL |
| *ATR* | c.6350A>G, p.(N2117S) | 0.0004 | - | 7 | GH |
| *ATRX* | c.1448A>C, p.(Q483P) | 0.009 | - | 8 | PRL |
| *ATRX* | c.2887G>C, p.(D963H) | 0 | - | 9 | NFA |
| *ATRX* | c.3559A>G, p.(N1187D) | 0.005 | - | 10 | PRL |
| *AXL* | c.1580G>A, p.(R527Q) | 0.002 | - | 11 | ACTH |
| *AXL* | c.2311C>A, p.(P771T) | 0.003 | - | 12 | GH |
| *AXL* | c.2498C>G, p.(P833R) | 0.003 | - | 13 | GH |
| *AXL* | c.541G>T, p.(A181S) | 0.02 | - | 14 | GH |
| *BCOR* | c.5234A>C, p.(H1745P) | 0.005 | - | 15 | GH |
| *BMP4* | c.668C>T, p.(R223H) | 0.01 | Hypospadias [4] and cleft palate [5] | 16 | Gn |
| *BMP4* | c.676C>T, p.(R226W) | 0.03 | Spina bifida [6] | 17 | NFA |
| *BMP4* | c.709G>A, p.(E237K) | 0.0007 | - | 18 | GH |
| *CHD7* | c.1028C>G, p.(S343C) | 0.0008 | - | 19 | PRL |
| *CHD7* | c.1046A>G, p.(N349S) | 0.009 | - | 20 | PRL |
| *CHD7* | c.1363A>G, p.(M455V) | 0.0004 | - | 21 | PRL |
| *CHD7* | c.2743G>A, p.(D915N) | 0 | - | 8 | PRL |
| *CHD7* | c.6248C>T, p.(P2083L) | 0.002 | - | 2 | PRL |
| *CHD7* | c.7471C>T, p.(R2491C) | 0.002 | - | 22 | GH |
| *CREBBP* | c.2348T>C, p.(M783T) | 0.0011 | - | 23 | PRL |
| *CREBBP* | c.2482G>T, p.(A828S) | 0.0180 | - | 4 | ACTH |
| *CREBBP* | c.3239C>T, p.(P1080L) | 0.0042 | - | 24 | PRL |
| *CREBBP* | c.4240G>A, p.(V1414I) | 0.0088 | - | 25 | PRL |
| *EP300* | c.1150T>G, p.(S384A) | 0.005 | - | 26 | GH |
| *EP300* | c.5878A>G, p.(T1960A) | 0 | - | 27 | ACTH |
| *EP300* | c.7157A>G, p.(H2386R) | 0.001 | - | 28 | PRL |
| *FGF8* | c.130C>T, p.(R44W) | 0.01 | Holoprosencephaly [7] | 29 | ACTH |
| *FGF8* | c.688C>T, p.(R230C) | 0.002 | - | 30 | GH |
| *FGFR1* | c.1447C>T, p.(P483S) | 0.0004 | Septo-optic dysplasia [8] | 31 | GH |
| *FGFR1* | c.386A>C, p.(D129A) | 0.014 | Kallmann syndrome [9] | 32, 33 | PRL, PRL |
| *FGFR2* | c.34G>A, p.(V12M) | 0.03 | - | 34 | ACTH |
| *FLNA* | c.448A>C, p.(I150L) | 0 | - | 35 | NFA |
| *FLNA* | c.901C>T, p.(R301W) | 0.006 | - | 36 | GH |
| *GATA2* | c.829A>G, p.(S277G) | 0.0110 | - | 37 | GH |
| *GATA2* | c.1027A>GCCA, p.(Ala342_Arg343insAla) | 0 | - | 15 | PH |
| *GLI2* | c. 3839A>G, p.(N1280S) | 0.001 | - | 38 | GH |
| *IGSF1* | c.1080C>A, p.(D360E) | 0 | - | 39 | PRL |
| *KAL1* | c.409C>G, p.(P137A) | 0 | - | 14 | GH |
| *LRP2* | c.2422A>G, p.(I808V) | 0.0020 | - | 14, 40 | GH, TSH |
| *LRP2* | c.2813G>A, p.(R938Q) | 0.0127 | - | 41 | GH |
| *LRP2* | c.2987G>A, p.(R996L) | 0.0111 | - | 42 | GH |
| *LRP2* | c.3266G>A, p.(R1089H) | 0.0371 | - | 43 | ACTH |
| *LRP2* | c.5113G>C, p.(A1705P) | 0.0004 | - | 34 | ACTH |
| *LRP2* | c.5368C>G, p.(Q1790E) | 0.0008 | - | 44 | PRL |
| *LRP2* | c.8341A>C, p.(N2781H) | 0.0007 | - | 45 | GH |
| *LRP2* | c.8452+2T>A | 0 | - | 19 | PRL |
| *LRP2* | c.10910C>T, p.(P3637L) | 0.0149 | - | 46 | GH |
| *LRP2* | c.13366C>T, p.(P4456S) | 0.0008 | - | 47 | NFA |
| *MED12* | c.2128G>A, p.(V710M) | 0.0011 | - | 48 | NFA |
| *OTX2* | c.641C>A, p.(T214A) | 0.02 | - | 30 | ACTH |
| *PAX5* | c.478T>C, p.(S160P) | 0.0004 | Bone marrow failure [10] | 16 | Gn |
| *PAX6* | c.590G>C, p.(G197A) | 0.004 | - | 22 | GH |
| *PAX7* | c.601G>A, p.(E201K) | 0 | - | 49 | PRL |
| *PAX7* | c.755A>G, p.(Q252R) | 0 | - | 9 | NFA |
| *PROP1* | c.193C>T, p.(R65C) | 0.001 | - | 50 | ACTH |
| *PTCH1* | c.324A>G, p.(I108M) | 0.0089 | Kallmann syndrome [11] | 51 | PRL |
| *PTCH1* | c.3391G>A, p.(V1131M) | 0.0024 | - | 52 | TSH |
| *PTCH1* | c.3422C>T, p.(A1141V) | 0.0036 | Gorlin syndrome [12] | 53 | GH |
| *SHH* | c.967G>A, p.(G323R) | 0 | - | 39 | PRL |
| *SUFU* | c.994C>G, p.(Q332E) | 0.0008 | - | 25 | PRL |
| *TCF7L1* | c.776T>A, p.(M259K) | 0 | - | 54 | GH |

*Abbreviations—*GH: growth hormone secreting tumour, PRL: prolactin-secreting tumour, NF: non-functioning tumour, ACTH: Adrenocorticotropic hormone-secreting tumour, TSH: thyroid stimulating hormone-secreting tumour, Gn: gonadotropin-secreting tumour, PH; plurihormonal tumour (expressing two or more pituitary hormones), F: familial cohort, S: sporadic cohort

**References**

1. McKusick-Nathans Institute of Genetic Medicine. Online Mendelian Inheritance in Man, OMIM® John Hopkins University, Baltimore [Available from: <https://omim.org/>.

2. Stenson PD, Mort M, Ball EV, Chapman M, Evans K, Azevedo L, et al. The Human Gene Mutation Database (HGMD®): optimizing its use in a clinical diagnostic or research setting. Human Genetics. 2020;139(10):1197-207. doi: 10.1007/s00439-020-02199-3.

3. Landrum MJ, Lee JM, Benson M, Brown GR, Chao C, Chitipiralla S, et al. ClinVar: improving access to variant interpretations and supporting evidence. Nucleic Acids Research. 2018;46(D1):D1062-D7. doi: 10.1093/nar/gkx1153.

4. Chen T, Li Q, Xu J, Ding K, Wang Y, Wang W, et al. Mutation screening of BMP4, BMP7, HOXA4 and HOXB6 genes in Chinese patients with hypospadias. European Journal of Human Genetics. 2007;15(1):23-8. doi: 10.1038/sj.ejhg.5201722.

5. Marini NJ, Asrani K, Yang W, Rine J, Shaw GM. Accumulation of rare coding variants in genes implicated in risk of human cleft lip with or without cleft palate. American Journal of Medical Genetics Part A. 2019;179(7):1260-9. doi: <https://doi.org/10.1002/ajmg.a.61183>.

6. Felder B, Stegmann K, Schultealbert A, Geller F, Strehl E, Ermert A, et al. Evaluation of BMP4 and its specific inhibitor NOG as candidates in human neural tube defects (NTDs). European Journal of Human Genetics. 2002;10(11):753-6. doi: 10.1038/sj.ejhg.5200875.

7. Hong S, Hu P, Roessler E, Hu T, Muenke M. Loss-of-function mutations in FGF8 can be independent risk factors for holoprosencephaly. Human Molecular Genetics. 2018;27(11):1989-98. doi: 10.1093/hmg/ddy106.

8. Raivio T, Avbelj M, McCabe MJ, Romero CJ, Dwyer AA, Tommiska J, et al. Genetic Overlap in Kallmann Syndrome, Combined Pituitary Hormone Deficiency, and Septo-Optic Dysplasia. The Journal of Clinical Endocrinology & Metabolism. 2012;97(4):E694-E9. doi: 10.1210/jc.2011-2938.

9. Albuisson J, Pêcheux C, Carel J-C, Lacombe D, Leheup B, Lapuzina P, et al. Kallmann syndrome: 14 novel mutations in KAL1 and FGFR1 (KAL2). Human Mutation. 2005;25(1):98-9. doi: <https://doi.org/10.1002/humu.9298>.

10. Bluteau O, Sebert M, Leblanc T, Peffault de Latour R, Quentin S, Lainey E, et al. A landscape of germ line mutations in a cohort of inherited bone marrow failure patients. Blood. 2018;131(7):717-32. doi: 10.1182/blood-2017-09-806489.

11. Barraud S, Delemer B, Poirsier-Violle C, Bouligand J, Mérol JC, Grange F, et al. Congenital Hypogonadotropic Hypogonadism with Anosmia and Gorlin Features Caused by a PTCH1 Mutation Reveals a New Candidate Gene for Kallmann Syndrome. Neuroendocrinology. 2021;111(1-2):99-114. doi: 10.1159/000506640.

12. Pruvost-Balland C, Gorry P, Boutet N, Magnaldo T, Mamelle G, Margulis A, et al. [Clinical and genetic study in 22 patients with basal cell nevus syndrome]. Ann Dermatol Venereol. 2006;133(2):117-23. doi: 10.1016/s0151-9638(06)70861-4.
